# Blockade of bone morphogenetic protein signaling potentiates the pro-inflammatory phenotype induced by interleukin-17 and tumor necrosis factor-α combination in rheumatoid synoviocytes

**DOI:** 10.1186/s13075-015-0710-6

**Published:** 2015-07-28

**Authors:** Alberto Varas, Jaris Valencia, Fabien Lavocat, Víctor G. Martínez, Ndiémé Ndongo Thiam, Laura Hidalgo, Lidia M. Fernández-Sevilla, Rosa Sacedón, Angeles Vicente, Pierre Miossec

**Affiliations:** Department of Cell Biology, Faculty of Medicine, Complutense University, Plaza Ramón y Cajal s/n, Madrid, 28040 Spain; Immunogenomics and Inflammation Research Unit and Department of Clinical Immunology and Rheumatology, Hospices Civils de Lyon, EA 4130 University of Lyon 1, Hôpital Edouard Herriot, Lyon, 69437 France

## Abstract

**Introduction:**

Bone morphogenetic proteins (BMPs) are multifunctional secreted growth factors regulating a broad spectrum of functions in numerous systems. An increased expression and production of specific BMPs have been described in the rheumatoid arthritis (RA) synovium. The aim of this study was to analyze the involvement of the BMP signaling pathway in RA synoviocytes in response to interleukin-17 (IL-17) and tumor necrosis factor-alpha (TNF-α).

**Methods:**

The expression of components of the BMP signaling pathway (BMP receptors, BMP ligands, BMP signal transducers, and BMP antagonists) was analyzed by quantitative polymerase chain reaction before and after treatment of RA synoviocytes with TNF-α or IL-17 or both. Regulation was studied in the presence of the specific BMP inhibitor DMH1 (dorsomorphin homologue 1) or an exogenous BMP ligand, BMP6. Expression and production of pro-inflammatory cytokines (IL-6 and granulocyte-macrophage colony-stimulating factor), chemokines (IL-8, CCL2, CCL5, and CXCL10), and matrix metalloproteinases (MMP-1, −2, −3, −9, and −13) were analyzed.

**Results:**

RA synoviocytes express BMP receptors (mainly BMPRIA, ACTRIA, and BMPRII), signal transducers of the Smad family (Smad1 and 5 and co-Smad4), and different BMP antagonists. The modulation of the expression of the BMP target genes—Id (inhibitor of DNA-binding/differentiation) proteins and Runx (Runt-related transcription factor) transcription factors—after the addition of exogenous BMP shows that the BMP signaling pathway is active. RA synoviocytes also express BMP ligands (BMP2, BMP6, and BMP7) which are highly upregulated after activation with TNF-α and IL-17. Autocrine BMP signaling pathway can be blocked by treatment with the inhibitor DMH1, leading to an increase in the upregulated expression of pro-inflammatory cytokines, chemokines, and MMPs induced by the activation of RA synoviocytes with TNF-α and IL-17. Conversely, the additional stimulation of the BMP pathway with the exogenous addition of the BMP6 ligand decreases the expression of those pro-inflammatory and pro-destructive factors.

**Conclusion:**

The results indicate that the canonical BMP pathway is functionally active in human RA synoviocytes and that the inhibition of autocrine BMP signaling exacerbates the pro-inflammatory phenotype induced in RA synoviocytes by the stimulation with IL-17 and TNF-α.

## Introduction

Bone morphogenetic proteins (BMPs) are secreted signaling proteins which form a subgroup of the transforming growth factor-beta (TGF-β) superfamily [1]. BMPs are dimeric proteins which, once secreted, bind to type I and type II BMP receptors constituting multimeric receptor-ligand complexes. Type II receptors are constitutively active serine/threonine kinases which trans-phosphorylate type I receptors upon ligand binding; subsequently, activated type I receptors phosphorylate and activate some components of the Smad protein family, Smad1, 5, and 8, called BMP receptor-regulated Smads (BR-Smads) [1–3]. The common mediator Smad4 next binds to BR-Smads, and the heteromeric complexes translocate to the nucleus to regulate the transcription of BMP target genes, including Id (inhibitor of DNA-binding/differentiation) proteins and Runx (Runt-related transcription factor) transcription factors [1, 2]. In addition to this canonical signaling pathway, activated BMP receptors may initiate non-canonical Smad-independent signaling pathways [1].

BMPs were originally identified as growth and differentiation factors for osteogenic cells but now are considered multifunctional proteins implicated in the development of virtually all organs and the renewal and maintenance of different adult tissues [1, 4–6]. The relevance of this pathway is further emphasized by the fact that an aberrant BMP signaling can result in several developmental defects and distinct human disorders, including cancer, chronic kidney diseases, endocrine alterations, vascular diseases, and joint and musculoskeletal disorders [7–10]. Rheumatoid arthritis (RA) is the most common form of chronic inflammatory arthritis characterized by persistent synovial inflammation, articular damage, and altered immune response [11]. Several BMP ligands, including BMP2, BMP6, and BMP7, have been shown to be upregulated in the synovium of patients with RA as well as in tumor necrosis factor-alpha (TNF-α) transgenic mice developing arthritis and in collagen-induced arthritis models [12–14]. High levels of BMP7 have also been demonstrated in the synovial fluid of patients with RA, and levels are correlated with severity of disease [15]. In contrast, BMP4 and BMP5 ligands are downregulated in the RA synovium [16]. In collagen-induced arthritis, a dynamic activation of the BMP signaling pathway has been reported, showing a time-dependent increase of the amount of phosphorylated BR-Smads and the number of phospho-Smad1/5/8-positive cells [13]. Furthermore, fibroblast-like synoviocytes from patients with RA have been demonstrated to express BMP receptors [17] and to upregulate the expression of BMP2 and mainly BMP6 after stimulation with pro-inflammatory cytokines such as TNF-α, interleukin-1beta (IL-1β), and IL-17 [14, 18, 19].

Despite all these data, however, only a few studies have addressed the involvement of BMP signaling in RA, pointing out a role for BMP6 and BMP7 in stimulating the survival and the proliferation and extracellular matrix component biosynthesis, respectively, in synoviocytes [12, 14]. In the present study, we show that the canonical BMP pathway is functionally active in human RA synoviocytes and that autocrine BMP signaling modulates the expression of pro-inflammatory cytokines, chemokines, and matrix metalloproteinases (MMPs).

## Methods

### Isolation and culture of rheumatoid arthritis synoviocytes

Synoviocytes were grown from synovial tissue samples obtained from RA patients undergoing joint surgery. The patients with RA fulfilled the American College of Rheumatology criteria for RA [20]. Each individual signed an informed consent form, and the protocol was approved by the committee for the protection of persons participating in biomedical research of the Hospital of Lyon, in compliance with the Helsinki Declaration. Briefly, synovial tissue was minced in small pieces which were allowed to fix on plastic plates. Those samples were maintained in Dulbecco’s modified Eagle’s medium (Eurobio, Courtaboeuf, France) supplemented with 10 % fetal bovine serum (Life Technologies, part of Thermo Fisher Scientific, Carlsbad, CA, USA), 2 % Penicillin-Streptomycin (Eurobio), 1 % L-glutamine (Eurobio), and 1 % Amphotericin B (Eurobio) until cells grew out of the tissue and colonized the plastic dishes. After cells reached confluence, tissue pieces were removed and cells were trypsinized. Synoviocytes were used between passages 4 and 9. Each independent experiment was performed with a different batch of synoviocytes isolated from different RA donors.

### RNA extraction and purification

Cells were seeded in 12-well plates at a density of 5×10^4^ cells/cm^2^. Cells then were treated for 12 h with different combinations of IL-17 50 ng/mL (R&D Systems, Minneapolis, MN, USA), TNF-α 0.5 ng/mL (R&D Systems), BMP-6 10 ng/mL (HumanZyme, Chicago, IL, USA), and dorsomorphin homologue 1 (DMH1) 30 μM (Tocris Bioscience, Ellisville, MO, USA). After 12-h treatment, RNA was extracted by using an RNeasy Plus Mini Kit (Qiagen, Venlo, Limburg, The Netherlands) in accordance with the instructions of the manufacturer. RNA then was quantified by using a Qubit RNA BR Assay Kit (Life Technologies).

### Real-time quantitative reverse transcription-polymerase chain reaction

Total cDNA was synthesized by using a High Capacity cDNA Reverse Transcription Kit (Applied Biosystems, Waltham, MA, USA) in accordance with the instructions of the supplier and then used as a target in the polymerase chain reaction (PCR) amplifications. Pre-designed TaqMan gene expression assays for different genes were obtained from Applied Biosystems. GNB2L1 was used as an endogenous control. All PCRs were set in duplicates by using the TaqMan Gene Expression Master Mix (Applied Biosystems) in accordance with the instructions of the manufacturer. Amplifications, detections, and analyses were performed in a 7.900HT Fast Real-time PCR System (Centro de Genómica, Complutense University, Madrid, Spain). The ∆Ct method was used for normalization to GNB2L1.

### Cytokine measurements

Culture supernatants of RA synoviocytes were harvested after 12 h of treatment. IL-8 secretion was measured by using an enzyme-linked immunosorbent assay (BioLegend, San Diego, CA, USA) and IL-6 and granulocyte-macrophage colony-stimulating factor (GM-CSF) were measured by using Cytometric Bead Array assays (BD Biosciences, Franklin Lakes, NJ, USA) in accordance with the instructions of the manufacturers.

### Statistical analysis

The Mann–Whitney test was used to compare differences. Values of *P* ≤ 0.05 (*), *P* ≤ 0.01 (**), and *P* ≤ 0.001 (***) were considered to be statistically significant.

## Results

### Bone morphogenetic protein signaling pathway is functionally active in human rheumatoid arthritis synoviocytes

**Fig. 1:**
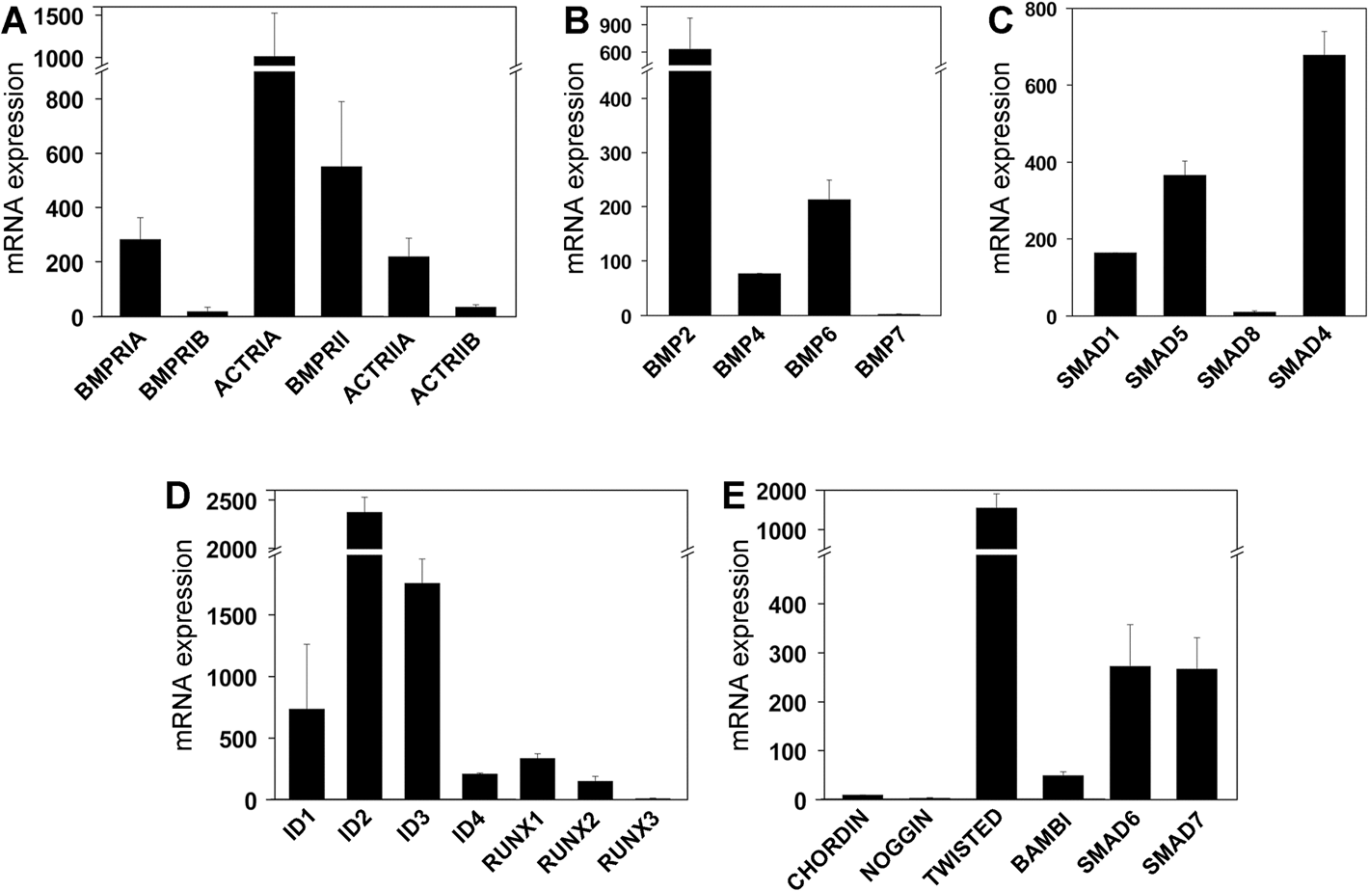
Expression of canonical BMP signaling components in RA synoviocytes. Synoviocytes grown from synovial tissue samples obtained from patients with RA were used between passages 4 and 9 to study the expression of several components of the canonical BMP signaling pathway by quantitative reverse transcription-polymerase chain reaction. Frames show the mRNA levels for **a** BMP receptors, **b** BMP ligands, **c** Smad protein family components, **d** BMP target genes, and **e** BMP antagonists. GNB2L1 was used as an endogenous control. Bars represent the mean (± standard deviation) of three to five independent experiments. *ACTRIA* type IA activin receptor, *ACTRIIA* type IIA activin receptor, *ACTRIIB* type IIB activin receptor, *BAMBI* bone morphogenetic protein and activin membrane-bound inhibitor, *BMP* bone morphogenetic protein, *BMPRIA* type IA bone morphogenetic protein receptor, *BMPRIB* type IB bone morphogenetic protein receptor, *BMPRII* type II bone morphogenetic protein receptor, *Id* inhibitor of DNA-binding/differentiation, *RA* rheumatoid arthritis, *Runx* Runt-related transcription factor, *Smad* small mother against decapentaplegic homolog

The expression of different components of the BMP signaling pathway was analyzed in RA synoviocytes. BMP receptor complexes are constituted by one of the following type I receptors, type IA BMP receptor (BMPRIA)/ALK-3, type IB BMP receptor (BMPRIB)/ALK-6, and type IA activin receptor (ACTRIA)/ALK-2, which commonly combine with type II BMP receptor (BMPRII) but also the type IIA and IIB activin receptors (ACTRIIA and ACTRIIB). In RA synoviocytes, BMPRIA and ACTRIA were the most abundantly expressed type I receptors, and the BMP-specific type II receptor BMPRII showed a high level of expression (Fig. 1a).

Among ligands, the most highly expressed were BMP2 and BMP6 (Fig. 1b). Synoviocytes also expressed specific RNAs for the Smad signal transducers, Smad1, Smad5, and the common partner Smad4, as well as the well-known BMP target genes, the Id protein family and Runx transcription factors [1] (Fig. 1c, d).

Furthermore, BMP signaling is finely regulated at multiple levels: at the extracellular space, several high-affinity antagonists bind selectively with BMPs to inhibit their biological actions; at the plasma membrane, the pseudoreceptor BAMBI associates with BMP receptors, preventing the formation of active receptor complexes; and intracellularly, inhibitory Smads and Smurfs inhibit further signaling and activation of target genes [21]. The expression of different BMP antagonists was also detected in RA synoviocytes, mainly the extracellular antagonist Twisted gastrulation and the intracellular inhibitors Smad6 and Smad7 (Fig. 1e). In contrast, only low levels of the pseudoreceptor BAMBI and the extracellular antagonists Noggin and Chordin could be detected in non-stimulated RA synoviocytes (Fig. 1e).

**Fig. 2:**
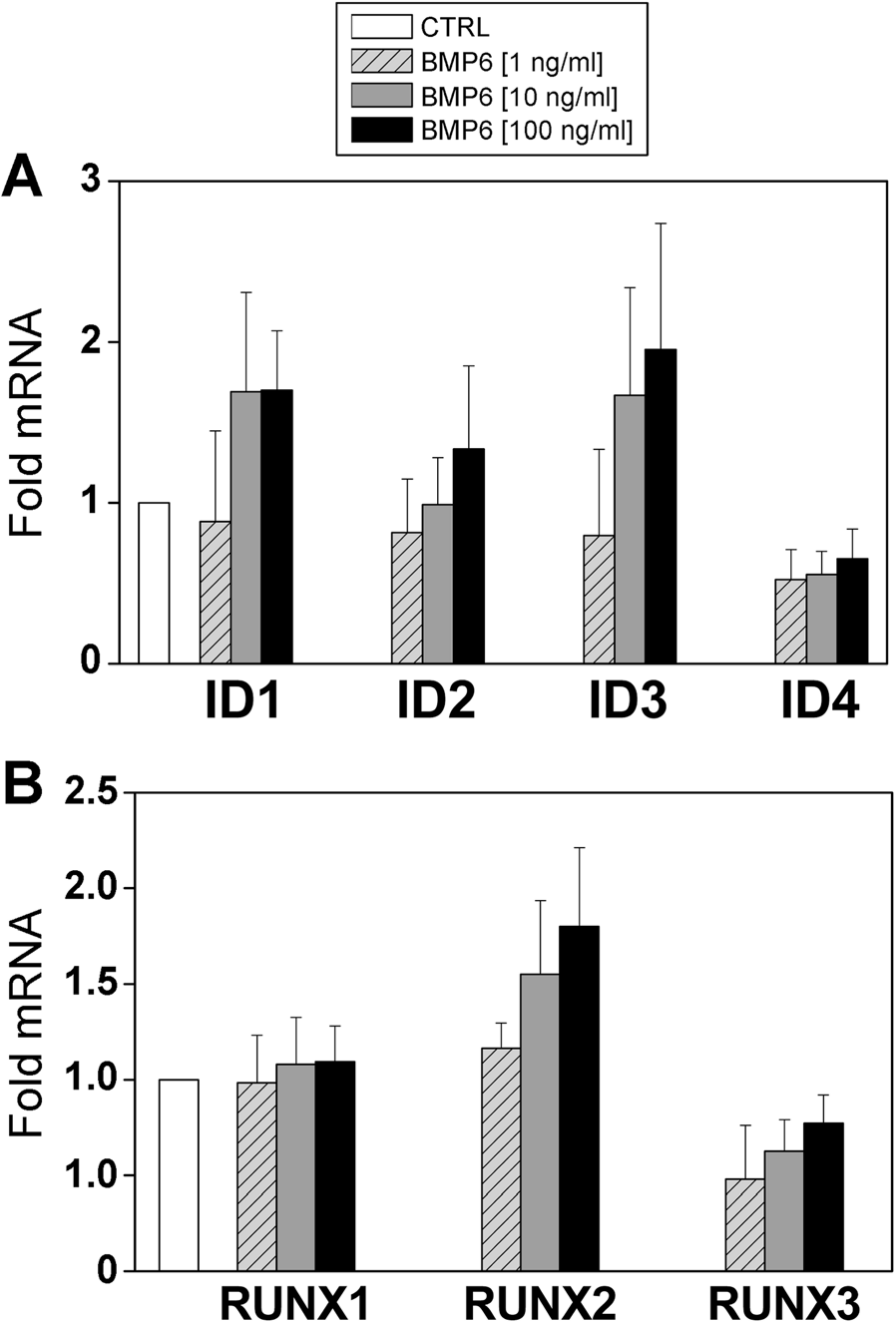
Modulation of BMP target gene expression in response to exogenous BMP stimulation. Synoviocytes from patients with rheumatoid arthritis were cultured in media alone (CTRL) or in the presence of increasing concentrations of BMP6 (1–100 ng/mL). After 12 h, cells were harvested and the expression of several target genes—Id1-4 **a** and Runx1-3 **b** factors—was analyzed by quantitative reverse transcription-polymerase chain reaction. GNB2L1 was used as an endogenous control. Results represent increments relative to cultures with media alone. The mean (± standard deviation) of three independent experiments is shown. *BMP* bone morphogenetic protein, *CTRL* control, *Id* inhibitor of DNA-binding/differentiation, *Runx* Runt-related transcription factor

The functionality of the BMP pathway was analyzed by studying the modulation of the expression of the components of Id protein and Runx families after addition of exogenous BMP. As shown in Fig. 2, stimulation of BMP signaling in synoviocytes showed a dose-dependent trend to increase the mRNA levels of Id1, Id2, and mainly Id3 and Runx2. On the contrary, the levels of transcripts for Id4 and Runx3 tended to decrease after BMP stimulation (Fig. 2a, b). These data indicated that BMP signaling pathway is functional in RA synoviocytes.

### Expression of bone morphogenetic protein pathway components is upregulated by pro-inflammatory cytokines in rheumatoid arthritis synoviocytes

**Fig. 3:**
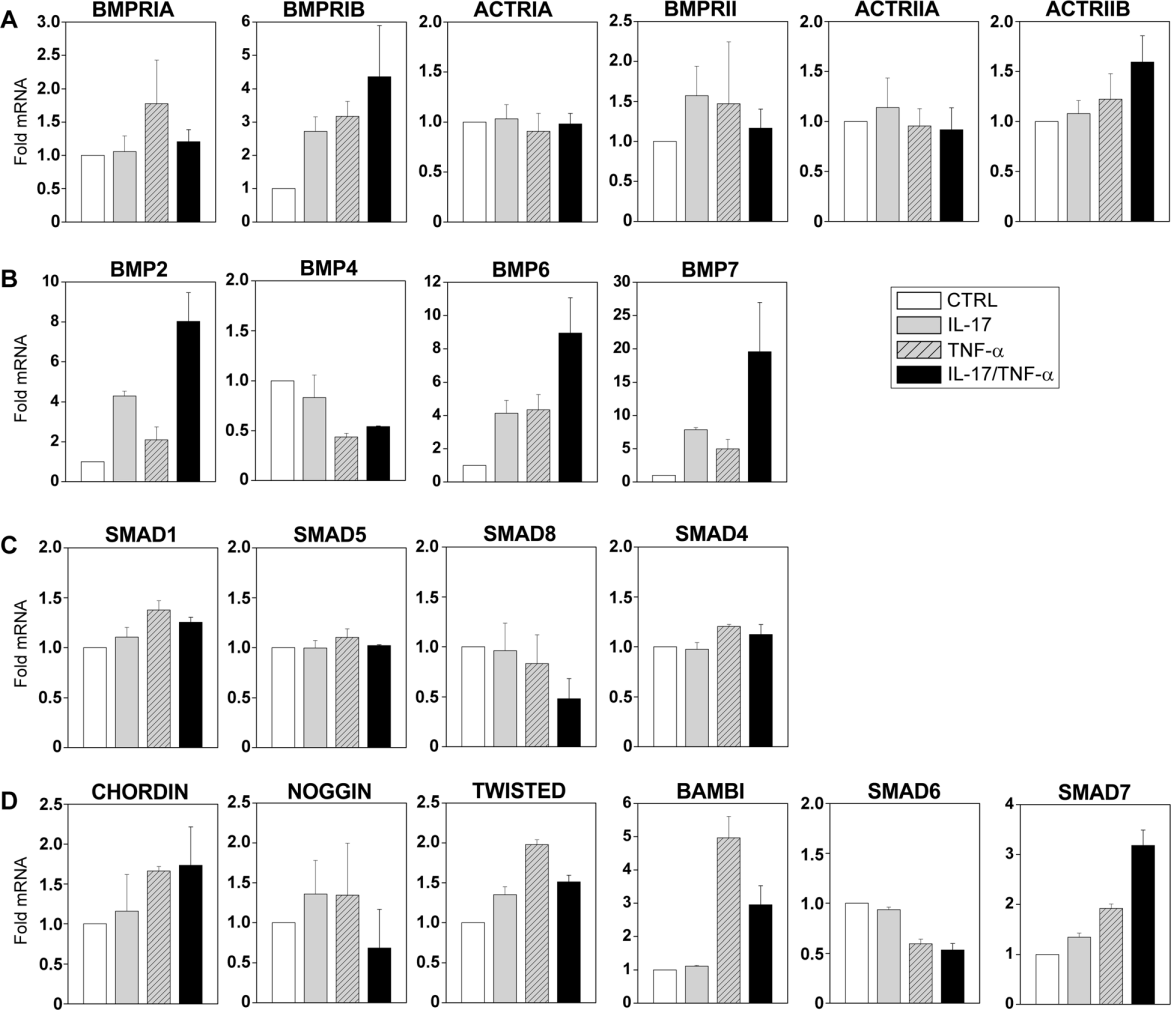
Regulation of BMP pathway components by pro-inflammatory cytokines. Rheumatoid arthritis synoviocytes were cultured in media alone (CTRL) or in the presence of IL-17 (50 ng/mL) or TNF-α (0.5 ng/mL) or both. **a-d** Expression of several BMP pathway components, including a BMP receptors, b BMP ligands, c Smad protein family components and d BMP antagonists, was studied by quantitative reverse transcription-polymerase chain reaction after 12 h of culture. GNB2L1 was used as an endogenous control. Results represent increments in respect to cultures in media alone. The mean (± standard deviation) of three independent experiments is shown. *ACTRIA* type IA activin receptor, *ACTRIIA* type IIA activin receptor, *ACTRIIB* type IIB activin receptor, *BAMBI* bone morphogenetic protein and activin membrane-bound inhibitor, *BMP* bone morphogenetic protein, *BMPRIA* type IA bone morphogenetic protein receptor, *BMPRIB* type IB bone morphogenetic protein receptor, *BMPRII* type II bone morphogenetic protein receptor, *CTRL* control, *IL-17* interleukin-17, *Smad* small mother against decapentaplegic homolog, *TNF-α* tumor necrosis factor-alpha

We next examined the effects of critical pro-inflammatory cytokines in RA, such as TNF-α and IL-17, on the expression of the different components of the BMP signaling pathway. The culture of synoviocytes with IL-17 and TNF-α alone or in combination induced mainly a 3- to 4-fold increase in the expression of BMPRIB, the least expressed type I BMP receptor in unstimulated synoviocytes, and slight increases in the mRNA levels of BMPRII and ACTRIIB receptors (Fig. 3a). The expression of Smad transcripts was hardly affected by the pro-inflammatory treatment. In contrast, the expression of BMP2, BMP6, and BMP7 ligands was notably increased after culture with IL-17 and TNF-α and mainly when the two cytokines were used in combination (8-, 9-, and 20-fold increases for BMP2, BMP6, and BMP7, respectively) (Fig. 3b, c). The levels of BMP4 mRNA were, however, reduced mainly in the presence of TNF-α (Fig. 3b). Interestingly, stimulation with TNF-α alone or with IL-17 enhanced the levels of transcripts encoding the membrane-bound antagonist BAMBI and the intracellular inhibitor Smad7 (3- to 5-fold and 2- to 3-fold increases, respectively) and, to a lesser extent, the extracellular modulator Twisted gastrulation (Fig. 3d).

### Blockade of canonical bone morphogenetic protein signaling potentiates cytokine production in rheumatoid arthritis synoviocytes

**Fig. 4:**
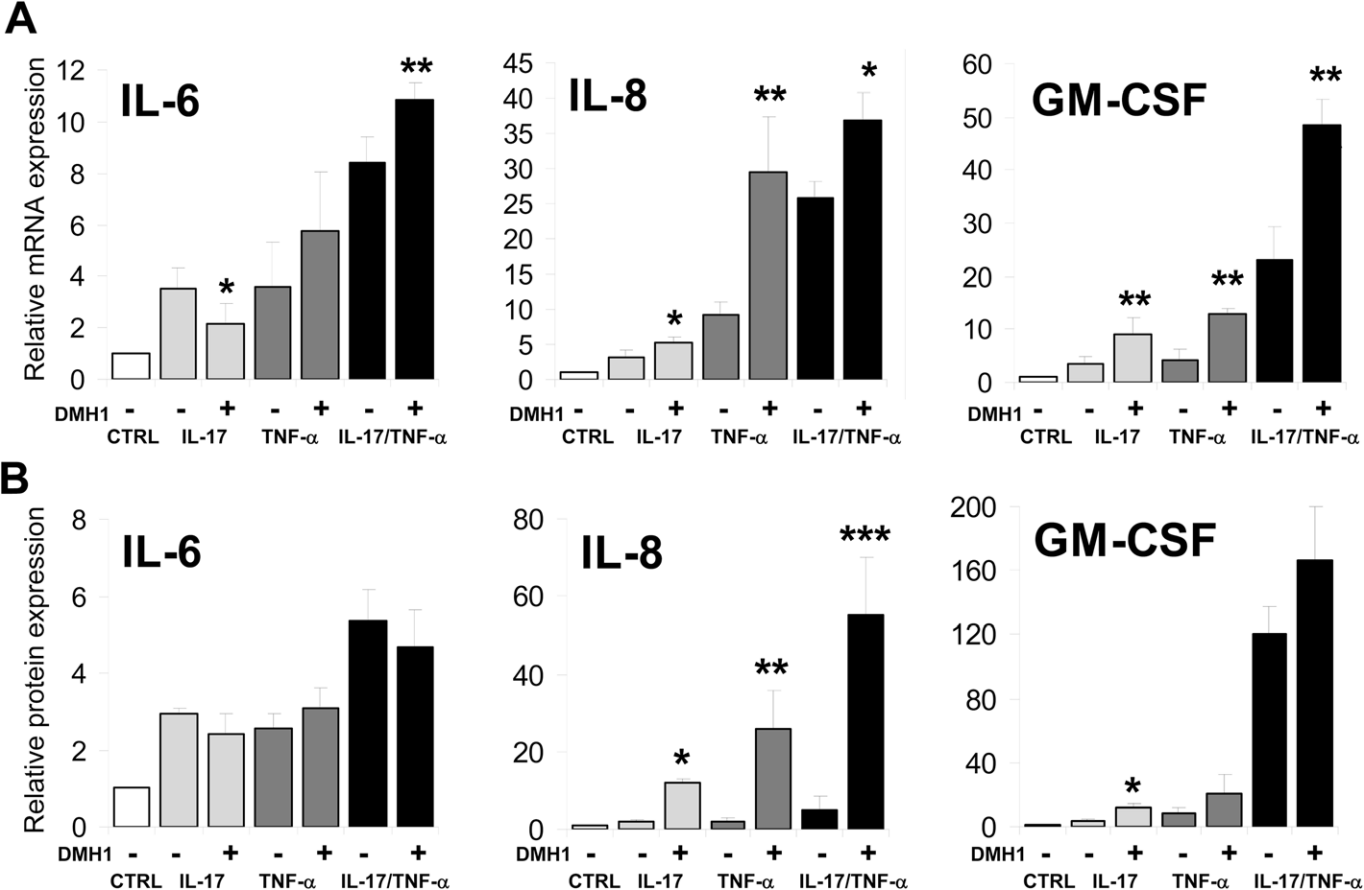
Effects of BMP signaling blockade on TNF-α- and IL-17-induced cytokine and chemokine mRNA and protein expression in RA synoviocytes. RA synoviocytes were simultaneously treated with the BMP pathway inhibitor DMH1 and IL-17 (50 ng/mL) or TNF-α (0.5 ng/mL) or both. **a** After 12 h, mRNA levels were analyzed by quantitative reverse transcription-polymerase chain reaction. Cells were left untreated as control, and the fold induction is shown for each treatment. GNB2L1 was used as an endogenous control. Bars represent the mean (± SD) of four to six independent experiments. Asterisks represent statistically significant differences between DMH1-treated and DMH1-non-treated cells (*P ≤0.05; **P ≤0.01; ***P ≤0.005; by Mann–Whitney test). **b** Protein levels were determined by enzyme-linked immunosorbent assay/Cytometric Bead Array assays. Data shown are expressed as fold induction compared with the untreated condition. Asterisks represent statistically significant differences between DMH1-treated and DMH1-non-treated cells (*P ≤0.05; **P ≤0.01; ***P ≤0.005; by *t* test). Bars represent the mean (± SD) of three to five independent experiments. *BMP* bone morphogenetic protein, *CTRL* control, *DMH1* dorsomorphin homologue 1, *GM-CSF* granulocyte-macrophage colony-stimulating factor, *IL* interleukin, *MMP* matrix metalloproteinase, *RA* rheumatoid arthritis, *SD* standard deviation, *TNF-α* tumor necrosis factor-alpha

The above data showing BMP receptor expression on synoviocytes, along with an upregulated BMP expression after pro-inflammatory cytokine stimulation, suggested that BMP ligands produced by RA synoviocytes could act in an autocrine manner. The highly selective type I BMP receptor inhibitor DMH1, which blocks the canonical BMP signaling pathway by inhibiting the BMP-induced Smad 1/5/8 activation [22], was used to study the role of endogenously produced BMP ligands in the expression of inflammatory factors in synoviocytes. As reported [18], IL-17, TNF-α, and mainly the combination of the two cytokines mostly upregulated the expression levels of the pro-inflammatory cytokines IL-6 and GM-CSF as well as the chemokine IL-8, all involved in RA pathogenesis (Fig. 4a). Remarkably, the simultaneous blockade of the canonical BMP signaling pathway induced a further upregulation of the expression of these genes, so that in some cases the higher levels reached after treatment with IL-17 or TNF-α or both underwent a further 2- to 3-fold increase (Fig. 4a). When protein levels were measured in the culture supernatants, the secretion of IL-8 and GM-CSF was also notably increased after treatment with the BMP inhibitor DMH1 (Fig. 4b). No significant variations were seen when IL-6 protein levels were determined (Fig. 4b). Therefore, these results suggested an anti-inflammatory role for BMP signaling.

### Modulation of chemokine and metalloproteinase expression in rheumatoid arthritis synoviocytes after canonical bone morphogenetic protein signaling inhibition

**Fig. 5:**
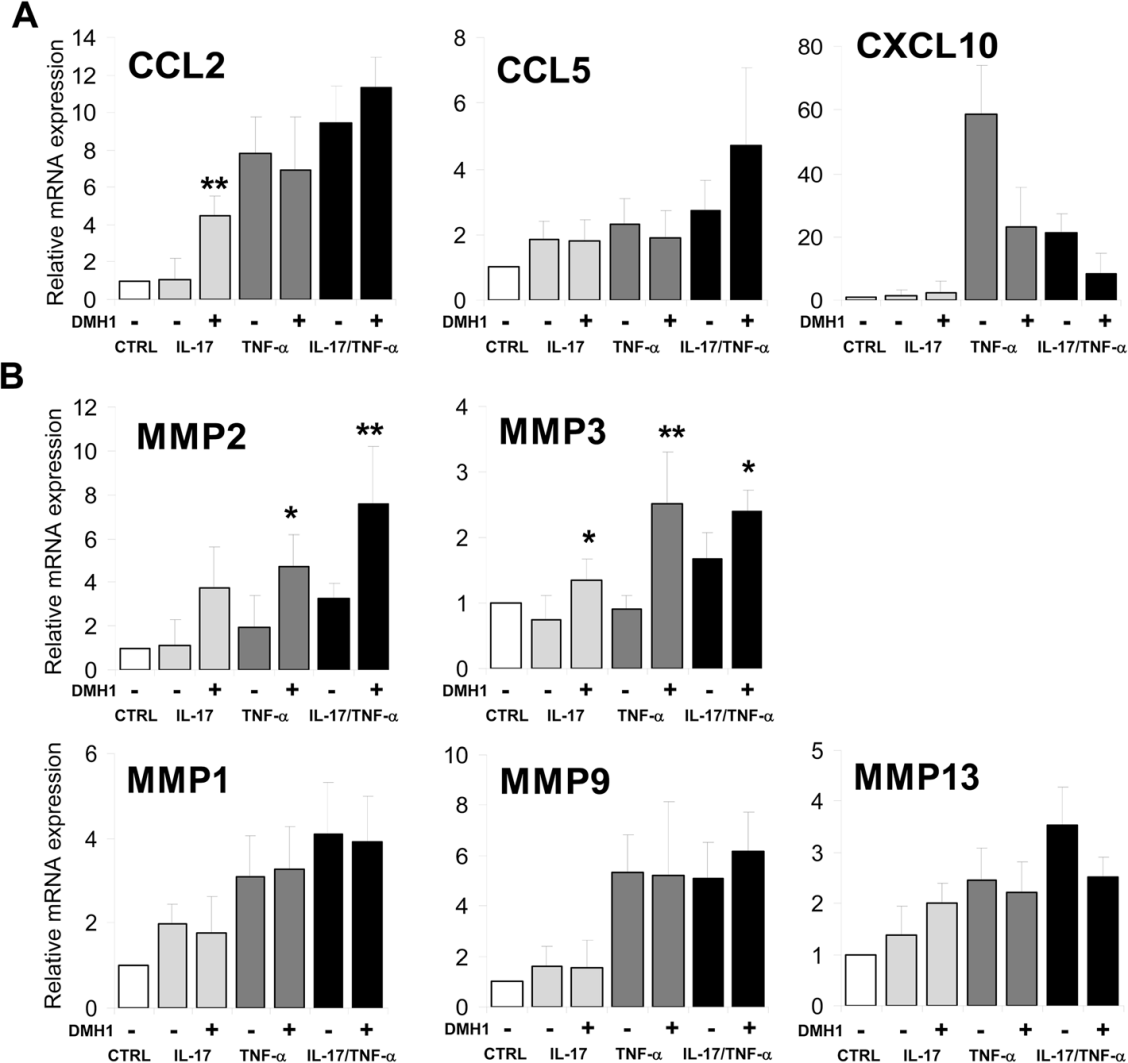
Effects of bone morphogenetic protein inhibitor DMH1 treatment on TNF-α and IL-17 combination-induced chemokine and matrix metalloproteinase mRNA expression in RA synoviocytes. Synoviocytes from patients with RA were left untreated (CTRL) or stimulated with IL-17 (50 ng/mL) or TNF-α (0.5 ng/mL) or both in the presence or absence of DMH1. The expression of **a** the indicated chemokines and **b** matrix metalloproteinases was analyzed by quantitative reverse transcription-polymerase chain reaction after 12 h of culture. GNB2L1 was used as an endogenous control. Fold induction relative to untreated cells is shown, and the mean (± standard deviation) of four to six independent experiments is presented. Asterisks represent statistically significant differences between DMH1-non-treated and DMH1-treated cells (*P ≤0.05; **P ≤0.01; ***P ≤0.005; by Mann–Whitney test). *CCL2* chemokine (C-C motif) ligand 2, *CCL5* chemokine (C-C motif) ligand 5, *CTRL* control, *CXCL10* chemokine (C-X-C motif) ligand 10, *DMH1* dorsomorphin homologue 1, *IL-17* interleukin-17, *MMP* matrix metalloproteinase, *RA* rheumatoid arthritis, *TNF-α* tumor necrosis factor-alpha

The expression of chemokines other than IL-8 was analyzed, and the results showed that BMP signaling inhibition mainly augmented the expression of CCL2 induced by IL-17 (Fig. 5a). However, the presence of the BMP inhibitor DMH1 did not significantly alter the expression of CCL2 and CCL5 or markedly reduced the high expression levels of CXCL10 induced in the presence of TNF-α alone or in combination with IL-17 (Fig. 5a).

The treatment of RA synoviocytes with IL-17 or TNF-α or both also upregulated the expression of MMP-2 and MMP-3 metalloproteinases involved in synoviocyte migration and invasion as well as joint destruction [18, 23] (Fig. 5b). When the canonical BMP signaling pathway was simultaneously inhibited by the addition of DMH1, the expression of both metalloproteinases underwent a further significant increase, mainly after treatment with TNF-α alone or together with IL-17 (Fig. 5b). In contrast, the upregulation of MMP-1, MMP-9, and MMP-13 metalloproteinase expression induced by IL-17 and TNF-α [24] was not significantly affected by the blockade of BMP signaling (Fig. 5b).

### Effects of the addition of BMP6 on cytokine, chemokine, and metalloproteinase expression in rheumatoid arthritis synoviocytes

**Fig. 6:**
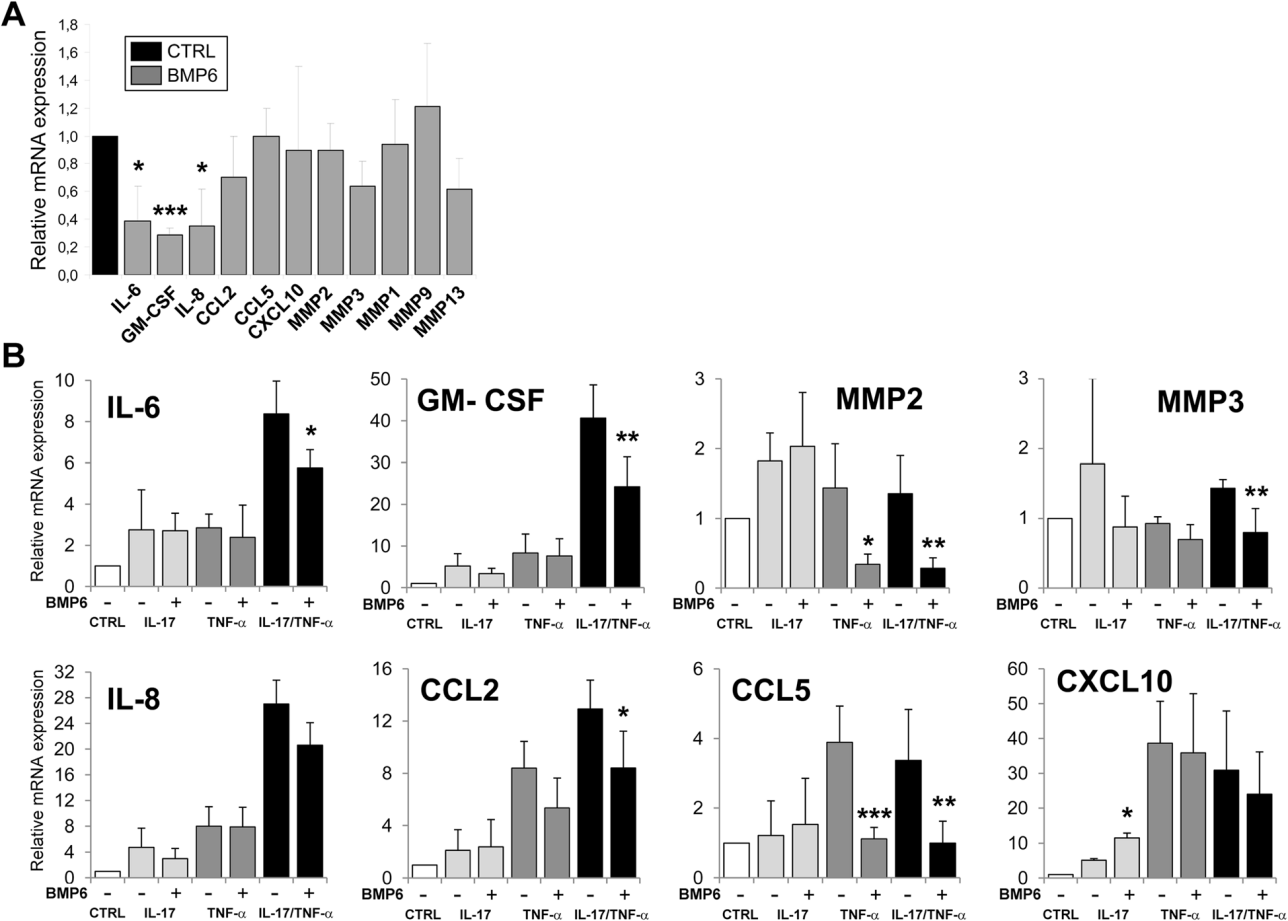
Effects of exogenous BMP6 stimulation on cytokine, chemokine, and matrix metalloproteinase expression in RA synoviocytes. **a** The expression of the indicated pro-inflammatory factors was analyzed by qRT-PCR in RA synoviocytes cultured in media alone (CTRL) or supplemented with BMP6 (10 ng/mL) for 12 h. **b** RA synoviocyte cultures were treated with IL-17 (50 ng/mL) or TNF-α (0.5 ng/mL) or both in the presence or absence of BMP6. After 12 h, mRNA levels for the indicated factors were analyzed by qRT-PCR. Cells were left untreated as control, and the fold induction is shown for each treatment. GNB2L1 was used as an endogenous control. Bars represent the mean (± standard deviation) of four to six independent experiments. Asterisks represent statistically significant differences between BMP6-treated and -non-treated cells (*P ≤0.05; **P ≤0.01; ***P ≤0.005; by Mann–Whitney test). *BMP* bone morphogenetic protein, *CCL2* chemokine (C-C motif) ligand 2, *CCL5* chemokine (C-C motif) ligand 5, *CTRL* control, *CXCL10* chemokine (C-X-C motif) ligand 10, *GM-CSF* granulocyte-macrophage colony-stimulating factor, *IL* interleukin, *MMP* matrix metalloproteinase, *qRT-PCR* quantitative reverse transcription-polymerase chain reaction, *RA* rheumatoid arthritis, *TNF-α* tumor necrosis factor-alpha

The effects of the addition of an exogenous BMP ligand, inducing an additional BMP signaling pathway activation, were also addressed. BMP6 was chosen because of its notable expression levels in RA synoviocytes and its high upregulation after synoviocyte stimulation with pro-inflammatory cytokines (Figs. 1b and 3b). Remarkably, in non-stimulated RA synoviocytes, cultured in the absence of IL-17 or TNF-α, the expression of IL-6, GM-CSF, and IL-8 was reduced by 50–70 % by the presence of BMP6, and to a lesser extent CCL2, MMP-3, and MMP-13 expression was reduced by 40 % (Fig. 6a). No relevant changes were observed in the expression of the metalloproteinases MMP-2, MMP-1, and MMP-9 and the chemokines CCL5 and CXCL10 (Fig. 6a). Meanwhile, in cytokine-activated RA synoviocytes, it could be noted that the further stimulation of BMP signaling tended to decrease the expression of most of the studied genes, mainly when TNF-α was present (Fig. 6b). No consistent differences were observed for the expression of MMP-1, MMP-9, and MMP-13 metalloproteinases after BMP ligand addition (not shown). All these data, then, supported the idea that the BMP pathway would have anti-inflammatory effects.

## Discussion

The increased expression and production of specific BMPs (i.e., BMP2, BMP6, and BMP7) in the synovium and synovial fluid of patients with RA as well as in mouse models of arthritis have been reported by different groups [12–15]. However, the involvement of the BMP signaling pathway in RA pathogenesis has been poorly analyzed.

In this study, we show the presence of a functionally active BMP signaling pathway in synoviocytes. Human RA synoviocytes express the three type I BMP receptors, BMPRIA, BMPRIB, and ACTRIA, and the BMP-specific type II receptor BMPRII. RA synoviocytes also express the BR-Smads as well as the common partner Smad4. All of these components are found to be efficient in signal transduction given the ability of synoviocytes to modulate the expression of some BMP target genes, including some Runx factors and members of the Id protein family, in response to exogenous BMP stimulation. In addition, RA synoviocytes express BMP ligands which, in agreement with previous work [14, 18, 19], are notably upregulated after stimulation with pro-inflammatory cytokines. These data suggest that some functional activities of synoviocytes could be modulated by BMP in an autocrine fashion, which is supported by our results using the selective BMP inhibitor DMH1.

**Fig. 7:**
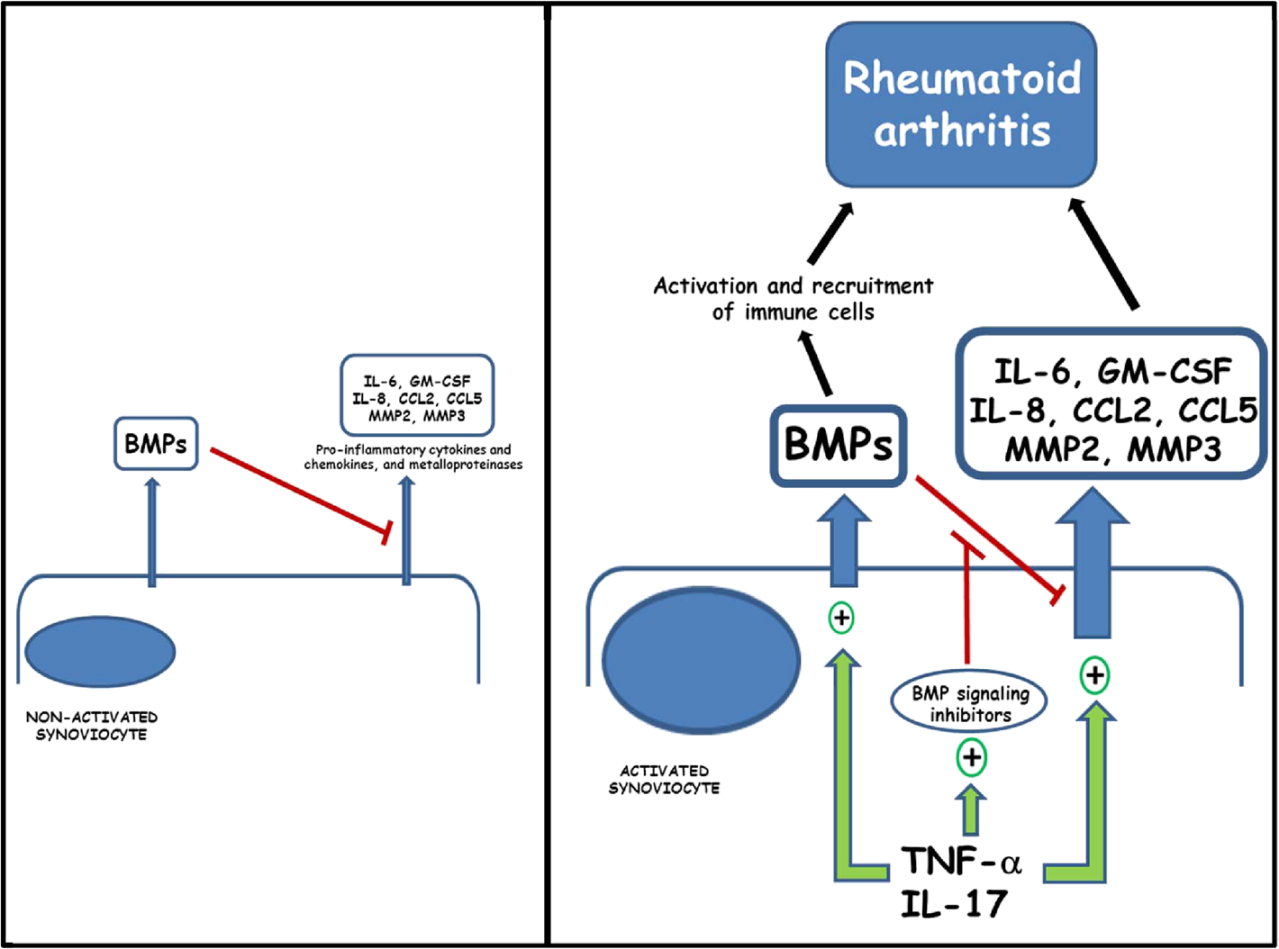
Schematic representation of BMP involvement in rheumatoid arthritis pathogenesis. Under steady-state conditions, autocrine BMP production could downregulate the expression and contribute to keep low levels of pro-inflammatory cytokines and chemokines as well as matrix metalloproteinases in synoviocytes. In the presence of increased levels of TNF-α and IL-17, synoviocytes become activated and enhance the production of BMPs which, however, could no longer control the levels of pro-inflammatory and pro-destructive factors because of the simultaneous upregulated expression of BMP signaling inhibitors in synoviocytes. Increased levels of BMPs could then participate in the recruitment and activation of immune cells contributing to rheumatoid arthritis along with the increased levels of pro-inflammatory cytokines, chemokines, and metalloproteinases. *BMP* bone morphogenetic protein, *CCL2* chemokine (C-C motif) ligand 2, *CCL5* chemokine (C-C motif) ligand 5, *GM-CSF* granulocyte-macrophage colony-stimulating factor, *IL* interleukin, *MMP* matrix metalloproteinase, *TNF-α* tumor necrosis factor-alpha

The blockade of BMP signaling with DMH1 significantly enhances the expression of pro-inflammatory cytokines (i.e., IL-6 and GM-CSF), chemokines (i.e., IL-8, CCL2, and CCL5), and MMPs (i.e., MMP-2 and MMP-3) induced in RA synoviocytes by the simultaneous treatment with IL-17 and TNF-α alone or in combination. Conversely, the stimulation of the BMP signaling pathway with an exogenous BMP ligand reduces the expression of pro-inflammatory and pro-destructive factors in both unstimulated and stimulated RA synoviocytes, mainly with the IL-17 and TNF-α combination. Therefore, it could be hypothesized that BMP signaling could have an anti-inflammatory role in the control and maintenance of low levels of pro-inflammatory factors in healthy synoviocytes and probably also in the early stages of RA (Fig. 7). In this regard, Lories and Luyten [25] also proposed that BMPs may play a disease-controlling role as joint-protective factors since BMP2 was described to promote synoviocyte apoptosis. Likewise, BMP7 has been shown to be able to suppress alterations in synoviocytes induced by synovial fluid from patients with RA, pointing out a role for BMP in maintaining a quiescent phenotype of the synovial lining layer [26].

However, the stimulation of RA synoviocytes induces the expression not only of BMP ligands but also of BMP antagonists, mainly after treatment with TNF-α alone or in combination with IL-17. Smad7 and BAMBI are the main upregulated BMP antagonists, which act at the intracellular and the plasma membrane level, respectively [21], indicating that BMP signaling inhibition must occur in the synoviocytes themselves. This finding suggests that in late stages of RA the BMP signaling pathway probably is no longer able to control and maintain the low levels of pro-inflammatory factors, which then will rise and persist at chronically high levels contributing to RA pathogenesis [27] (Fig. 7).

In this context, the high levels of BMP ligands produced by stimulated synoviocytes could also contribute to the chronic inflammation associated with RA by acting in a paracrine fashion on surrounding cells present in the inflamed synovium (Fig. 7). BMPs have been shown to induce a pro-inflammatory phenotype in endothelial cells [28, 29] and to stimulate chemotactic responses in monocytes/macrophages [30, 31], which play a central role in RA. BMP pathway activation increases monocyte adherence to endothelial cells [28] and stimulates the production of pro-inflammatory cytokines, including TNF-α, IL-1, and IL-6, by macrophages [32, 33]. We and others have shown that BMP stimulation induces maturation of dendritic cells [34], increases T-cell proliferation and activation [35, 36], and promotes T helper 17 (Th17) differentiation [36]. Furthermore, BMPs have been shown to be chemotactic for mesenchymal stem cells [37–39], which can expand Th17 cells, as we reported previously [40], and osteoclast differentiation and activity can also be enhanced by some BMP ligands [41], which could contribute to the bone destruction seen in RA.

## Conclusions

We propose that BMPs would play a dual role in the synovium, controlling the production of pro-inflammatory cytokines and inhibiting synoviocyte transformation in healthy and non-inflammatory conditions, but in advanced stages of RA could contribute to chronic inflammation, allowing the functional deregulation of synoviocytes and promoting the activation and pro-inflammatory functions of immune and non-immune cells accumulating in the inflamed arthritic joint.

## Abbreviations

ACTRIA: Type IA activin receptor
ACTRIIA: Type IIA activin receptor
ACTRIIB: Type IIB activin receptor
BAMBI: Bone morphogenetic protein and activin membrane-bound inhibitor
BMP: Bone morphogenetic protein
BMPRIA: Type IA bone morphogenetic protein receptor
BMPRIB: Type IB bone morphogenetic protein receptor
BR-Smad: Bone morphogenetic protein receptor-regulated Smad
CCL2: Chemokine (C-C motif) ligand 2
CCL5: Chemokine (C-C motif) ligand 5
CXCL10: Chemokine (C-X-C motif) ligand 10
DMH1: Dorsomorphin homologue 1
GM-CSF: Granulocyte-macrophage colony-stimulating factor
Id protein: Inhibitor of DNA-binding/differentiation protein
MMP: Matrix metalloproteinase
RA: Rheumatoid arthritis
Runx: Runt-related transcription factor
Smad: Small mother against decapentaplegic homolog
TGF: Transforming growth factor
Th17: T helper 17
TNF: Tumor necrosis factor
